# University of Louisville

**Published:** 1871-01

**Authors:** 


### UNIVERSITY OF LOUISVILLE·

We are pleased to learn of the prosperity of the Medical Department of this excellent Institution. Its present class embraces students from many States of the Union, and numbers two hundred and thirty. We wish our friends of the Medical Department an uninterrupted flow of success and prosperity.

We are informed that a few days since a young girl, living in the country, near our city, in anticipation of an event which is a common sequence of loving too well but not wisely, prepared a bed of straw and leaves in an old field, for the emergency. Urged by her instincts a few days subsequent to making these arrangements, she suddenly disappeared without notice—much to the alarm of her friends, and doubtless that of her lover. After diligent search, she was discovered in her premature bed—but not its sole occupant. Alone and unassisted this poor girl passed safely through one of the most trying ordeals incident to human life—performing for herself the office of accoucher.

Sam Patch made just one leap too often, below the Falls of Niagara, having previously safely accomplished the feat. We would advise an imitation neither of Patch's nor this reckless disregard of life

Our Correspondent—Student.—A communication will be found in our present number from one of our best and most gifted friends. For this reason, we highly appreciate its reception, and it will afford us great pleasure to publish it from the fact that it brings to us good news—the reunion of great and good men in a noble work, and of the great and merited prosperity of an old and favored School of Medicine; and the bright prospects and wide field for good and usefulness that lies before the talented and gifted young gentlemen of its faculty. It always affords us the sincerest pleasure to disseminate the truth, and at all times, to recognize and applaud true merit: "We chronicle their successes with hearty good will." "When God gives a man wings, he will fly, clip them as you may."

Our Thanks are due to one of our friends, Dr. C., for his kind advice in regard to our Journal. We deplore, with him, the evident lack of information on the part of Physicians in reference to the best business houses, who, but for the want of this information, would be able to satisfy all the demands of the profession, in every respect that he mentions. We hope, in a short time, to be able to lay before our readers the names of the first business houses of the country—those manafacturing chemicals and drugs; those making instruments, &c., and those publishing standard works on medical topics.

